# Optic Perineuritis Secondary to CNS Involvement of Lymphoma

**DOI:** 10.7759/cureus.75818

**Published:** 2024-12-16

**Authors:** Rohma R Khan, Abdul Mahmood, Sunny Kahlon, Steven A Benyahia

**Affiliations:** 1 Internal Medicine, University of South Florida, Tampa, USA; 2 Inpatient Pharmacy, Orlando Health, Orlando, USA

**Keywords:** blood-brain barrier (bbb), cns lymphoma, diffuse large b-cell lymphoma (dlbcl), disease progression, intrathecal chemotherapy, methotrexate, ommaya reservoir, optic neuritis, optic perineuritis, vision loss

## Abstract

CNS lymphoma is a rare form of non-Hodgkin lymphoma that primarily affects the brain, spinal cord, leptomeninges, or eyes, leading to severe neurological or ophthalmological complications. This case report details a 44-year-old male with human immunodeficiency virus and diffuse large B-cell lymphoma who experienced permanent vision loss due to optic perineuritis, a rare presenting symptom indicative of underlying CNS involvement. Despite previous remission, imaging revealed focal enhancements suggesting CNS lymphoma, highlighting diagnostic and management challenges in relapsed lymphoma, especially in immunocompromised patients. The patient's rapid symptom onset and subsequent irreversible vision loss emphasize the need for early detection and intervention. Despite aggressive treatment with systemic and intrathecal chemotherapy, the patient's visual function did not recover. This case highlights the importance of monitoring high-risk lymphoma patients for CNS relapse by way of regular neuroimaging to facilitate prompt diagnosis and treatment of optic nerve involvement, thereby mitigating the risk of irreversible neurological damage. Future research should focus on optimizing management strategies for CNS lymphoma and improving outcomes for patients with such rare and debilitating complications.

## Introduction

CNS lymphoma is a rare manifestation of non-Hodgkin lymphoma, comprising 4% of all CNS malignancies [1]. It primarily affects the brain, spinal cord, leptomeninges, or eyes, leading to significant neurological and ocular complications. The involvement of the optic nerve in CNS lymphoma, while extremely uncommon, can result in profound visual deficits, as this case demonstrates. Optic perineuritis, characterized by inflammation of the sheath surrounding the optic nerve, may serve as a presenting symptom of underlying CNS involvement, leading to often irreversible vision loss. In this case, we present a 44-year-old male with human immunodeficiency virus (HIV) and diffuse large B-cell lymphoma (DLBCL) who was previously in remission but developed gradual left eye vision loss secondary to optic perineuritis. Further workup revealed CNS lymphoma as the cause of his symptoms, highlighting the challenges of diagnosing and managing CNS relapse in lymphoma patients, particularly those with concurrent HIV infection. This case underscores the rarity of optic nerve involvement in lymphoma and the critical importance of early detection and treatment in preserving neurological function. 

## Case presentation

The patient is a 44-year-old male with a medical history notable for HIV and stage IV DLBCL. He presented with a primary complaint of vision loss in his left eye, having experienced a mild left temporal headache in the days prior. He described a gradual onset of central and inferior vision loss, which ultimately progressed to total vision loss (with no light perception remaining) over the course of four days. Additionally, he reported pain and swelling in his left eye. Upon arrival at the emergency department, he was promptly evaluated by the ophthalmology team. 

An ophthalmologic exam revealed edema of the optic nerve head, characterized by blurred disc margins and obscured vessels, with no evidence of exudate or hemorrhage; venous tortuosity was also observed. Computed tomography angiography (CTA) of the head and neck indicated no significant stenosis or occlusion in the intracranial or cervical vessels. A CT scan of the head showed no acute intracranial abnormalities. Magnetic resonance venography (MRV) of the brain, with and without contrast, revealed no signs of dural sinus thrombosis. Additionally, an MRI of the brain showed focal enhancement and mild edema in the left occipital cortex, along with focal enhancement of the left trigeminal nerve. 

**Figure 1:**
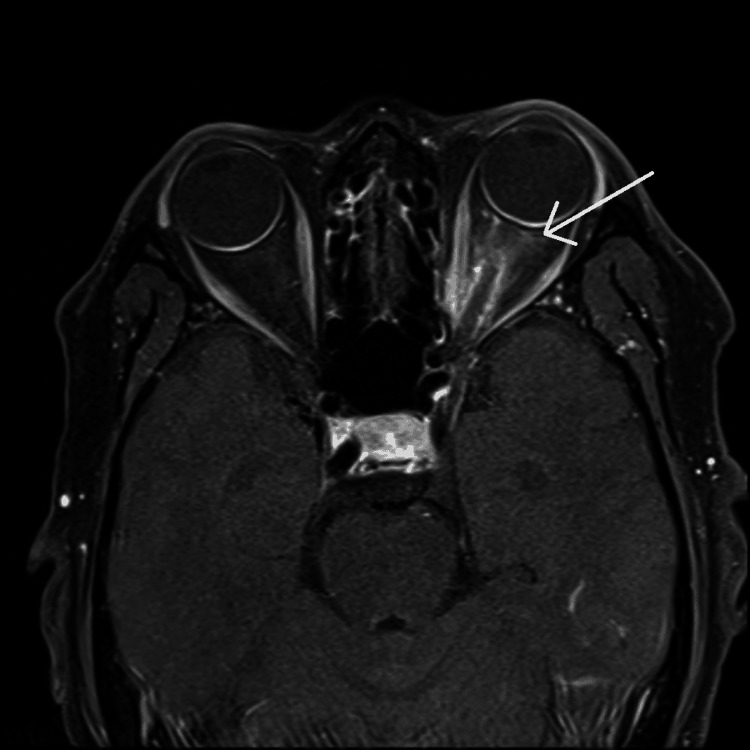
MRI of the orbits with and without contrast MRI, magnetic resonance imaging

The findings raised concerns for potential leptomeningeal involvement by the patient’s known lymphoma, though meningitis could present similarly. There was no evidence of acute infarction or intracranial hemorrhage. MRI of the orbits (with and without contrast), as seen in Figure 1, showed enhancement and edema involving the left optic nerve sheath without involvement of the optic nerve, consistent with optic perineuritis; the right orbit, as shown, is unremarkable. MRI of the cervical, thoracic, and lumbar spine (with and without contrast) did not reveal any signs of malignancy. 

At this point, the patient had already completed four cycles of DA-EPOCH-R along with four cycles of intrathecal cytarabine, spanning a total of three months. One month prior to the above symptoms occurring, he was considered to be in complete remission. The DA-EPOCH-R regimen includes etoposide phosphate, prednisone, vincristine sulfate (Oncovin), cyclophosphamide, doxorubicin hydrochloride (hydroxydaunorubicin), and rituximab, with "DA" referring to dose-adjusted. Throughout his chemotherapy, the patient continued taking prophylactic medications, including trimethoprim-sulfamethoxazole and acyclovir, along with his HIV regimen of bictegravir, emtricitabine, and tenofovir. 

Upon admission, the patient was found to have an undetectable HIV viral load and a cluster of differentiation 4 count of 195 cells/mm^3^. Basic laboratory tests, including a CBC and complete metabolic panel, were unremarkable. A lumbar puncture was performed, revealing CSF studies with lymphocytic predominance, raising concerns for CNS lymphoma. The CSF total protein was elevated, while CSF glucose levels were normal. The CNS meningitis panel, which included tests for cytomegalovirus, herpes simplex virus 1 and 2, varicella zoster virus, Epstein-Barr virus, *Escherichia coli*, enterovirus, human herpesvirus 6, *Haemophilus influenzae*, cryptococcus, BK virus, and John Cunningham virus, returned negative results. Additionally, treponemal testing, toxoplasma IgG, and Quantiferon tests were all normal (Table 1). 

**Table 1:** Laboratory values ALT, alanine transaminase; AST, aspartate aminotransferase; BUN, blood urea nitrogen; CD4, cluster of differentiation 4; CMV, cytomegalovirus; CSF, cerebrospinal fluid; EBV, Epstein-Barr virus; E. coli, Escherichia coli; HHV6, human herpesvirus 6; HIV, human immunodeficiency virus; H. influenzae, Haemophilus influenzae; HSV1, herpes simplex virus type 1; HSV2, herpes simplex virus type 2; IgG, immunoglobulin G; JC, John Cunningham; PCR, polymerase chain reaction; RBC, red blood cell; RNA, ribonucleic acid; SGOT, serum glutamic-oxaloacetic transaminase; SGPT, serum glutamic pyruvic transaminase; VZV, varicella-zoster virus; WBC, white blood cell

Parameters	Result	References
Sodium	139 mmol/L	135-148 mmol/L
Potassium	3.9 mmol/L	3.5-5.3 mmol/L
Chloride	100 mmol/L	98-107 mmol/L
CO_2_	22 meq/L	22-29 meq/L
BUN	14.0 mg/dL	8.9-20.6 mg/dL
Glucose	112 mg/dL	70-110 mg/dL
Calcium	10.70 mg/dL	8.40-10.20 mg/dL
Creatinine	1.0 mg/dL	0.7-1.3 mg/dL
AST (SGOT)	31.0 U/L	5.0-34.0 U/L
ALT (SGPT)	45 U/L	5-55 U/L
Total Bilirubin	0.3 mg/dL	0.2-1.2 mg/dL
Alkaline Phosphatase	235 U/L	40-150 U/L
Total Protein	8.8 g/dL	6.4-8.3 g/dL
Albumin	4.6 g/dL	3.5-5.0 g/dL
Anion Gap	17 meq/L	5-13 meq/L
WBC	11.78 x 10³ /µL	4.60-10.20 x 10³ /µL
Hemoglobin	12 g/dL	14.1-18.1 g/dL
Platelet Count	459 x 10³ /µL	142-424 x 10³ /µL
Absolute CD4 count	195 /uL	500-2,600 /uL
HIV RNA Quantitative PCR Test	<30 copies/mL	20-10,000,000 copies/mL
Treponemal Antibody Test	Nonreacted	Nonreactive
Toxoplasma IgG	Negative	Negative
Quantiferon	Negative	Negative
CSF Nucleated Cells	2 /uL	0-4 /uL
CSF RBC	0 /uL	0-0 /uL
CSF Neutrophils	0%	0%-6%
CSF Lymphocytes	81%	40%-80%
CSF Total Protein	56 mg/dL	15.0-45.0 mg/dL
CSF Glucose	66 mg/dL	40-70 mg/dL
CSF CMV	Not Detected	Not Detected
CSF HSV1	Not Detected	Not Detected
CSF HSV2	Not Detected	Not Detected
CSF VZV	Not Detected	Not Detected
CSF EBV	Not Detected	Not Detected
CSF *E. coli*	Not Detected	Not Detected
CSF Enterovirus	Not Detected	Not Detected
CSF HHV6	Not Detected	Not Detected
CSF *H. influenzae*	Not Detected	Not Detected
CSF Cryptococcus	Not Detected	Not Detected
CSF BK Virus	Not Detected	Not Detected
CSF JC Polyomavirus	Not Detected	Not Detected

The patient was started on a three-day course of high-dose IV solumedrol, leading to improvements in the pain and swelling of his left eye. Neurosurgery was consulted, and an Ommaya reservoir was placed for the intrathecal methotrexate. Due to a potential interaction with methotrexate, his trimethoprim-sulfamethoxazole was switched to atovaquone. He completed the first cycle of high-dose methotrexate, followed by intrathecal methotrexate, and was subsequently discharged with plans to continue chemotherapy on an outpatient basis. Unfortunately, his left eye vision loss remained unchanged at the time of discharge despite these interventions. 

## Discussion

Given the patient’s presentation, it was crucial to rule out vascular, neoplastic, and infectious causes. During the initial infectious disease consultation, the likelihood of an infectious etiology was deemed low for several reasons. The patient exhibited normal mentation, had no fever or chills, and did not report significant headache or neck pain/stiffness; this made meningitis unlikely. Additionally, his adherence to HIV treatment and undetectable viral load reduced the risk of opportunistic infections. He also presented without facial rash, which would raise concerns for varicella zoster virus. There were no signs of retinitis or uveitis to suggest other viral infections such as cytomegalovirus. Moreover, the abrupt onset of symptoms without fever pointed more toward ocular vessel occlusion, thrombosis, or tumor infiltration rather than infection. Vascular causes were effectively ruled out through negative results from CT of the head, CTA of the head and neck, and MRV of the brain. As a result, neoplastic etiology became the primary consideration in the differential diagnosis. 

The patient’s lymphoma relapsed after he was thought to have achieved complete remission. Studies suggest that DLBCL relapse occurs in approximately 30%-40% of patients, with CNS relapse being particularly challenging due to the blood-brain barrier, which complicates treatment delivery [2]. The patient underwent Ommaya reservoir implantation; this is an intraventricular catheter system that allows long-term access to the CSF for simplified administration of medication directly into the brain [3]. Intrathecal chemotherapy via the Ommaya reservoir, along with high-dose methotrexate, was used in this patient’s case to overcome the limitations posed by the blood-brain barrier. However, such treatments often carry a high risk of complications, and their efficacy in optic nerve involvement is still being debated [2]. 

Although the patient had been in remission, the recurrence of symptoms, particularly with CNS involvement, suggests that his lymphoma may have remained undetected for some time. This case underlines the need for vigilant long-term follow-up in patients with aggressive lymphomas and possibly even early CNS-directed therapy in certain high-risk populations, including patients with HIV. HIV’s link with DLBCL is well-established [4]. DLBCL is 15 times more common in people with HIV than in people without HIV, perhaps resulting from the T-cell dysfunction and impaired immunoregulation that HIV causes; DLBCL is also considered to be an AIDS-defining illness [5]. In this case, the patient had well-controlled HIV with an undetectable viral load, suggesting that the lymphoma's development might have been influenced by earlier periods of immune dysregulation or other factors, rather than ongoing immunosuppression. DLBCL is one of the most common malignancies in patients with HIV, and while antiretroviral therapy has improved outcomes, these patients remain at higher risk of both developing lymphoma and facing aggressive disease courses, including CNS involvement [4]. Research shows that HIV-positive patients with DLBCL tend to have poorer prognoses compared to their HIV-negative counterparts, largely due to immune system limitations in managing malignancies and infections [4]. This case exemplifies the complex interplay between HIV and lymphoma, where even well-controlled HIV may not fully mitigate cancer risks. 

While CNS lymphoma can affect various parts of the brain, its direct involvement with the optic nerve leading to vision loss is unusual [6,7]. This rarity poses a challenge in both diagnosis and treatment, as the involvement of such a delicate structure limits therapeutic options [8]. This makes aggressive treatment critical but also carries the risk of long-term visual impairment, as seen in this case. Given the rare presentation, it is worth exploring documented cases of optic nerve involvement in lymphoma, their outcomes, and the extent to which vision can be preserved or restored following aggressive therapy. 

Unfortunately, the patient’s vision did not improve despite treatment with high-dose steroids and chemotherapy; this could have been attributed to several factors. The optic nerve, once damaged by inflammation or direct neoplastic infiltration, may not fully recover, especially if there is prolonged compression, ischemia, or demyelination [9,10]. In this patient, the relatively rapid onset of symptoms coupled with delayed presentation to the hospital resulted in irreversible damage. Studies indicate that CNS lymphoma involving the optic nerve has a poor prognosis for visual recovery, and in this case, despite maximal therapeutic efforts, the loss was permanent [11]. 

Beyond the optic nerve, lymphoma can cause a range of ocular complications, including vitreous infiltration, uveitis, and retinal involvement [12]. In this case, there was no evidence of these complications, but the sudden visual loss suggests the optic nerve was an early target of the relapse, possibly related to perineural spread or direct infiltration. This case raises questions about how to improve early detection, including the potential for regular neuroimaging in high-risk patients or those with concerning symptoms such as visual changes. Since relapses are more common in the first two years of treatment, CT scans can be completed every six months for surveillance during this time [13].

## Conclusions

This case highlights the complexities of diagnosing and managing CNS lymphoma, particularly in patients with a history of DLBCL and HIV. The rare involvement of the optic nerve, leading to significant vision loss, underscores the need for heightened awareness among healthcare providers regarding potential CNS relapse in lymphoma patients. The interplay between HIV and lymphoma complicates clinical outcomes, as patients with well-controlled HIV still face increased risks for aggressive malignancies such as DLBCL. The challenges of effective treatment delivery across the blood-brain barrier necessitate innovative approaches, such as intrathecal therapy via an Ommaya reservoir. However, as seen in this case, even aggressive interventions may not restore vision once significant neuro-ophthalmic damage has occurred. This case serves as a reminder of the importance of early detection and prompt intervention in high-risk populations. Regular neuroimaging and vigilant follow-up are essential strategies to prevent irreversible complications from CNS involvement. Future research should focus on refining management protocols for CNS lymphoma and improving outcomes for patients with optic nerve involvement, thereby enhancing our understanding of this rare yet impactful manifestation of lymphoma.
